# Prevalence of betel nut chewing and its pattern of distribution across the Bangladeshi socio-demographics

**DOI:** 10.1038/s41598-025-01460-x

**Published:** 2025-07-16

**Authors:** Abrar Wahab, Koustuv Dalal, Minhazul Abedin, Shagoofa Rakhshanda, Farah Naz Rahman, A. K. M. Fazlur Rahman, Aminur Rahman, Saidur Rahman Mashreky

**Affiliations:** 1 Centre for Injury Prevention and Research Bangladesh (CIPRB), Dhaka, Bangladesh; 2 Division of Public Health Science, Department of Health Sciences, Mid Sweden University, Sundsvall, Sweden; 3 Department of Population Health Science, John D. Bower School of Population Health, University of Mississippi Medical Center, Jackson, MS USA; 4 International Centre for Diarrhoeal Disease Research (ICDDR,B), Dhaka, Bangladesh; 5 Department of Public Health, North South University, Dhaka, Bangladesh

**Keywords:** Addiction, Betel nut, Prevalence, Correlates, Socio-demographics, Risk factors, Cancer

## Abstract

In Bangladesh, chewing betel nut has become culturally and socially approved, becoming a mounting public health concern. This study aimed to estimate the current prevalence of betel nut chewing, identify the sociodemographic correlates in urban and rural settings, and identify any vulnerable group for future policy-making to address this problem. This is a retrospective study of secondary data analysis based on the data of the Bangladesh Health and Injury Survey 2016. The outcome variable of interest was the self-reported practice of chewing betel nuts. Only ≥ 18 years old participants were considered. In the logistic regression model, the odds ratios with 95% confidence intervals were used to determine the correlates of each independent variable. The multivariable model’s explanatory variables were all tested to ensure no multi-collinearity in the final model. The prevalence of betel nut chewing was 31.4% among the study population. Multivariate analysis showed that participants aged 65 years and above were 10 times more likely to be betel nut users (AOR 10.17; 95% CI 9.58–10.79), while rural populations were 1.24 times more likely to use betel nut (AOR 1.24; 95% CI 1.21–1.27). Given the significant public health burden, targeted interventions are warranted to mitigate its impact. The findings indicate that betel nut chewing is highly prevalent, particularly among older, illiterate, and rural populations, demonstrating stark social and geographic inequalities. However, as the data were collected in 2016, the current socio-political and economic changes in Bangladesh may have influenced this trend, limiting the direct applicability of the findings to the present context.

## Introduction

Around 10% of the world’s population, about 600 million people, consume betel leaf in conjunction with areca nut^1^. The most conventional form of consuming a betel leaf (leaf of piper betel) is wrapping it with some areca nut anointed with slaked lime (calcium hydroxide). Most of the time, the betel leaf consumer consumes it with tobacco and other flavouring agents^2^. After consuming it a few times, tobacco soon became the most commonly used ingredient with the betel quid^3^. Chewing betel nut is a common custom integrated into social and cultural practices and ceremonies^4^. The less fortunate people of the community can afford it since it is a less expensive pleasure. It is mainly practised as an indigenous ritual and religious custom among people residing within Asia Pacific, South and South-East Asia^5^. Various kinds of formulations are used from region to region. Hence its colloquial name differed along with geography.

The high prevalence of betel nut chewing raises serious public health concerns due to its association with oral cancer and oral potentially malignant disorders (OPMD). In Sri Lanka, betel nut use is linked to a high incidence of oral squamous cell carcinoma, with disproportionate effects on lower socioeconomic groups^6^. It also increases the risk of many chronic non-communicable diseases (NCDs) such as Hypertension, Metabolic syndrome, Diabetes mellitus, Cardiovascular diseases etc.^7–11^. Areca nut has also been associated with a lower appetite, deteriorated digestion, altered attention, anaemia, pregnancy complications, relaxation, and decreased alertness in some cases^12–14^. Understanding these risks is crucial for designing effective intervention strategies.

Betel nut chewing has become a mounting public health concern in Bangladesh. In Bangladesh, constituents of betel nut include areca nut (known as “Supari” in Bengali) along with betel leaf (known as “Pan” in Bengali) and slaked lime (known as “Chun” in Bengali), termed altogether as "Pan Chewing/Betel Nut Chewing”. A substantial number of the population indulged in this practice, not knowing its deleterious effects on health. A national survey targeting specifically male respondents showed that around 17.5% of 15–54 years older men consumed betel nut^15^. Another study conducted among rural married women in 2011 found that 25% of the study participants were “currently consuming” smokeless tobacco^16^.

Despite its widespread consumption, nationally representative studies on betel nut chewing in Bangladesh remain scarce. Prior research has largely focused on specific subgroups rather than providing a comprehensive epidemiological overview. Additionally, existing studies have not adequately explored how betel nut use varies across different sociodemographic and geographic settings. This study addresses these gaps by analyzing a large, nationally representative dataset, allowing for a more precise identification of at-risk groups.

Moreover, Bangladesh lacks a coordinated policy framework to mitigate betel nut consumption. Unlike neighboring South Asian countries, where public health campaigns and policy measures have been implemented, Bangladesh has few structured interventions targeting this issue.

Thus, this study will be highly relevant in understanding the current trend of betel nut chewers in rural and urban settings. The findings of this study provide a critical foundation for designing context-specific interventions and informing evidence-based policy strategies to reduce betel nut use.

## Materials and methods

This study used a retrospective secondary data analysis based on the Bangladesh Health and Injury Survey data (BHIS, 2016). Bangladesh Health and Injury Survey 2016 was a community-based, nationally representative cross-sectional survey conducted between March and June 2016. The survey utilized a multistage cluster sampling technique with discrete urban and rural samples based on probability-proportion-to-size (PPS) methods. Among 64 districts of Bangladesh, 16 were randomly selected. From each district, one Upazila was randomly selected for the rural area. The village was considered a cluster, and 100 villages were selected from each Upazila, whereas 80 households were selected from each. A systematic sampling technique was adopted for the urban area to attain the required number of households from mohallas/wards (clusters). Three hundred fifty thousand respondents from 70,000 households were targeted to be sampled during the study. After cleaning and validation, data from 299,216 household residents were available for analysis^17^. Sampling biases were minimized by following complex sampling strategies that use probability-proportional-to-size methods (PPS). In this survey, Tablets were utilized to collect data with the help of a custom data entry program called REDCAP. Data were uploaded to the server and downloaded for later cleaning and manipulation. The original study was approved by the Ethical Review Committee of the Centre for Injury Prevention and Research Bangladesh (CIPRB).

The outcome variable of interest was self-reported chewing of betel nuts. Participants self-reported their current betel nut consumption. Those who responded affirmatively to consuming betel nut at least once a week were classified as betel nut users. The participants in this secondary analysis were the adult population ≥ 18 years only. After selecting participants with ≥ 18 years old, the total number of participants for this current study was 195,482. The independent variables of interest were sex, age categorized as 18–24, 25–44, 45–64, and over 65 years old, educational level of the participants, marital status, occupation, geographical location of residence (urban/rural), and self-reported status of smoking. Cases with missing responses on betel nut consumption or key demographic variables were excluded from the final analysis to minimize bias.

The analysis was performed using the Statistical Package for the Social Sciences (SPSS) version 23. Descriptive statistics of the explanatory variables were presented in frequencies and percentages with their 95% confidence interval (CI). In univariate and multivariate logistic regression models, the odds ratio with 95% CI was used to observe any association between the independent and outcome variables. A value of *p* ≤ 0.05 was considered statistically significant. To confirm that the final model did not have multi-collinearity, all independent variables of the multivariate model were assessed before performing multivariate logistic regression.

This study used publicly available secondary data from BHIS-2016, which was ethically approved by the Centre for Injury Prevention and Research, Bangladesh. No additional ethical approval was required for secondary analysis as per institutional guidelines. All methods were carried out in accordance with the Ethical Committees’ guidelines and regulations based on Helsinki Declarations. BHIS-2016 confirmed that informed consent was obtained from all participants as stipulated by the Ethical Committees guidelines.

## Results

### Characteristics of the study population

There were 195,482 study participants with a mean age of 38.4 years (range 18–116 years). Table 1 shows us that the majority of the study population was in the age group of 25–44 years at 50.2% (98,174), followed by the age group of 45–64 years at 23.9% (46,697), 18–24 years at 18.9% (37,040) and lastly ≥ 65 years at 6.9% (13,571). The study’s female population comprised 50.9% (99,484), and the rest were male at 49.1%. Almost two-thirds of the population, 63.3% (123,817), were rural, whereas the remaining 36.7% were from urban areas.

**Table 1:** Population characteristics and prevalence of betel nut chewer among the study population (n = 195,482).

Variables	Population (n)	Total		Urban		Rural	
		Prevalence (%)	95% CI	Prevalence (%)	95% CI	Prevalence (%)	95% CI
Sex							
Male	95,998 (49.1%)	32,296 (33.6%)	33.3–33.9	9934 (28.2%)	27.6–28.5	22,362 (36.8%)	36.4–37.1
Female	99,484 (50.9%)	29,113 (29.3%)	28.9–29.5	8664 (23.8%)	23.3–24.2	20,449 (32.4%)	32.0–32.8
Age							
18–24 years	37,040 (18.9%)	2518 (6.8%)	6.5–7.0	759 (5.4%)	5.0–5.7	1759 (7.7%)	7.3–8.0
25–44 years	98,174 (50.2%)	26,457 (26.9%)	26.6–27.2	8373 (22.3%)	21.8–22.7	18,084 (29.8%)	29.4–30.2
45–64 years	46,697 (23.9%)	24,719 (52.9%)	52.4–53.3	7592 (46.5%)	45.7–47.2	17,127 (56.4%)	55.8–56.9
≥ 65 years	13,571 (6.9%)	7715 (56.8%)	56.0–57.6	1874 (51.2%)	49.5–52.8	5841 (58.9%)	57.9–59.9
Residence							
Urban	71,665 (36.7%)	18,598 (26.0%)	25.6–26.2	–	–	–	–
Rural	123,817 (63.3%)	42,811 (34.6%)	34.3–34.8	–	–	–	–
Education							
Illiterate	30,664 (15.7%)	13,358 (43.6%)	43.0–44.1	2715 (37.7%)	36.5–38.8	10,643(45.4%)	44.7–46.0
1–5 years of schooling	55,128 (28.2%)	21,801 (39.5%	39.1–39.9	6390 (37.3%)	36.5–37.9	15,411(40.6%)	40.0–41.0
6–12 years of schooling	80,762 (41.3%)	16,436 (20.4%)	20.0–20.6	7205 (20.0%)	19.5–20.3	9231(20.7%)	20.2–21.0
> 12 years of schooling	14,154 (7.2%)	1285 (9.1%)	8.6–9.5	790 (9.2%)	8.6–9.8	495 (8.9%)	8.1–9.6
Informal education (Maktab)	14,774 (7.6%)	8529 (57.7%)	56.9–58.5	1498 (56.4%)	54.5–58.3	7031 (58.0%)	57.1–58.8
Occupation							
Business	24,876 (12.7%)	8814 (35.4%)	34.8–36.0	4119 (33.2%)	32.3–34.0	4695 (37.7%)	36.8–38.5
Service Holder	17,561 (9.0%)	2951 (16.8%)	16.2–17.3	1690 (15.8%)	15.1–16.5	1261 (18.4%)	17.4–19.2
Agriculture	20,674 (10.6%)	9351 (45.2%)	44.5–45.9	341 (46.1%)	42.5–49.8	9010 (45.2%)	44.5–45.8
Skilled/Unskilled Labor	26,839 (13.7%)	10,437 (38.9%)	38.3–39.4	3853 (37.6%)	36.7–38.5	6584 (39.7%)	38.6–40.1
Housewife	84,723 (43.3%)	26,020 (30.7%)	30.4–31.0	7510 (25.0%)	24.5–25.5	18,510 (33.8%)	33.4–34.2
Unemployed/Retired	20,809 (10.6%)	3836 (18.4%)	17.9–19.0	1085 (14.3%)	13.5 – 15.1	2751 (20.8%)	20.1 – 21.5
Marital Status							
Never Married	21,086 (10.8%)	1138 (5.4%)	5.0–5.7	287 (3.6%)	3.1–4.0	851 (6.5%)	6.1–6.9
Ever Married	174,396 (89.2%)	60,271 (34.6%)	34.3–34.7	18,311 (28.8%)	28.4–29.1	41,960 (37.9%)	37.6–38.1
Smoking Habit							
Smoker	42,175 (21.6%)	19,819 (47.0%)	46.5–47.4	6137 (39.9%)	39.1–40.6	13,682 (51.1%)	50.4–51.6
Non-Smoker	153,307 (78.4%)	41,590 (27.1%)	26.9–27.3	12,461 (22.1%)	21.7–22.4	29,129 (30.0%)	29.7–30.3

Almost half of the study population (80,762) had a formal education of 6–12 years of schooling, whereas 28.2% (55,128) had 1–5 years of schooling, 15.7% (30,664) of the study population were illiterates. Only 7.2% (14,154) of the population had more than 12 years of schooling. We have also found that 7.6% (14,774) of the population had an informal level of education, commonly known as “Maktab”, in Bangladesh. Among the study population, Service, business, agriculture, labour, and unemployed/retired comprised 56.7% of the participants, and the rest, 43.3%, were homemakers. Most (89.2%, 174,396) of the participants were ever married. Current smokers made up 21.6% (42,175) of the participants, and the rest, 78.4% (152,307) the participants, were non-smokers (Table 1).

### Prevalence of betel nut among the participants

Table 1 shows that 33.6% (32,296) and 29.3% (29,113) of males and females were found to be betel nut users. Betel nut chewing practices increase with the increasing age of 25 years onwards. The highest amount of betel nut users was found among the “Informal education (Maktab)” and “No literacy” groups, 57.7% (8529) and 43.6% (13,358), respectively. Almost half of the participants with agriculture occupations, 45.2% (9351), were found to be betel nut users, followed by skilled/unskilled labour 38.9% (10,437), business owners 35.4% (8814), and homemakers 30.7% (26,020). Among the current smokers 47% (19,819) and non-smokers 27.1% (41,590) were betel nut chewers (Table 1).

We also looked at the frequency of betel nut consumption in urban and rural populations. In our analysis, we have found that 28.2% (9934) of the urban male had betel nut chewing practices, whereas 36.8% (22,362) were among the rural male population. A similar trend of betel nut chewing practice was also observed among the urban (23.8%) and rural (32.4%) female populations. Across the different age spectrums of the urban population, the proportion of betel nut chewing was 5.4% at 18–24 years, 22.3% at 25–44 years, 46.5% at 45–64 years, and 51.2% at ≥ 65 years. The rural population follows a similar pattern of an increase in the proportion of betel nut chewing with the gradual increase of age but with a higher prevalence rate, such as 7.7% at 18–24 years, 29.8% at 25–44 years, 56.4% at 45–64 years, and 58.9% at ≥ 65 years. The proportion of betel nut chewing among the urban population with no literacy was found to be 37.7%, whereas 45.4% among the rural population with no literacy. Although betel nut chewing was more prevalent among rural populations than urban populations, the percentage of participants who chewed betel nuts was similar for those with 6–12 years of schooling. The betel nut chewing rate was almost nine per cent higher between urban homemakers (25.0%) and rural housewives (33.8%). It was also observed that the prevalence of betel nut chewing was almost 10% higher among the ever-married group of people living in a rural area than the ever-married group of people living in an urban area. Among the rural smoker population, 51.1% were betel nut chewers, whereas the proportion of betel nut chewers among urban smokers was 39.9% (Table 1).

### Correlates of betel nut chewing practice

The univariate analysis found that males, older age, rural residency, illiteracy, and smoking are significantly associated with chewing betel nuts (Table 2).

**Table 2:** Univariate and multivariate logistic regression analysis with correlates of chewing betel nut.

Variables	Univariate analysis			Multivariate analysis		
	SE	OR (95% CI)	*p* value	SE	AOR (95% CI)	p value
Sex						
Male (Ref)	–	–	–	–	–	–
Female	0.01	0.82 (0.80 – 0.83)	< 0.001	0.03	0.96 (0.91 – 1.02)	0.162
Age						
18—24 years (Ref)		–	–	–		
25–44 years	0.02	5.06 (3.02–3.31)	< 0.001	0.02	3.18 (3.04–3.33)	< 0.001
45–64 years	0.02	15.42 (14.75–16.12)	< 0.001	0.03	8.35 (7.95–8.76)	< 0.001
≥ 65 years	0.03	18.06 (17.13–19.04)	< 0.001	0.03	10.17 (9.58–10.79)	< 0.001
Residence						
Urban (Ref)		–	–	–	–	–
Rural	0.01	1.51 (1.48–1.54)	< 0.001	0.01	1.24 (1.21–1.27)	< 0.001
Education						
More than 12 years of schooling (Ref)		–	–	–	–	–
Illiterate	0.03	7.73 (7.27–8.22)	< 0.001	0.04	2.82 (2.63–3.03)	< 0.001
1–5 years of schooling	0.03	6.55 (6.17–9.96)	< 0.001	0.03	3.43 (3.21–3.67)	< 0.001
6–12 years of schooling	0.03	2.56 (2.41–2.72)	< 0.001	0.03	1.90 (1.78–2.03)	< 0.001
Informal education (Maktab)	0.03	13.68 (12.80–14.61)	< 0.001	0.04	5.06 (4.69–5.45)	< 0.001
Occupation						
Service (Ref)		–	–	–		
Agriculture	0.03	4.09 (3.90–4.29)	< 0.001	0.03	1.32 (1.25–1.40)	< 0.001
Unemployment/Retired	0.03	1.12 (1.06–1.18)	< 0.001	0.04	1.10 (1.03–1.18)	0.004
Business	0.02	2.72 (2.59–2.85)	< 0.001	0.03	1.47 (1.40–1.55)	< 0.001
Skilled/Unskilled Labor	0.02	3.15 (3.01–3.30)	< 0.001	0.03	1.60 (1.53–1.70)	< 0.001
Housewife	0.02	2.19 (2.10–2.29)	< 0.001	0.03	1.53 (1.43–1.63)	< 0.001
Marital Status						
Never Married (Ref)		–	–	–		
Ever Married	0.03	9.26 (8.17–9.84)	< 0.001	0.04	2.35 (2.19–2.52)	< 0.001
Smoking Habit						
Non-smoker (Ref)		–	–	–		
Smoker	0.01	2.38 (2.33–2.44)	< 0.001	0.02	1.88 (1.82–1.93)	< 0.001

The reference group is betel nut non-chewer group.

According to the multivariate analysis (Table 2), the study participants who were 65 years and above were ten times ((AOR 10.17; 95% CI 9.58–10.79), 45–64 years were eight times (AOR 8.35; 95% CI 7.95–8.76), and 25–44 years are three times (AOR 3.18; 95% CI 3.04–3.33) more likely to be betel nut users than participants with an age range of 18–24 years old. The rural population were 1.24 times more likely to be betel nut users than the urban dwellers (AOR 1.24; 95% CI 1.21–1.27).

Those with no literacy, 1–5 years of schooling, and 6–12 years of schooling were more likely to be two times (AOR 2.82; 95% CI 2.63–3.03), three times (AOR 3.43; 95% CI 3.21–3.67), almost two times (AOR 1.90 (95% CI 1.78–2.03) current betel nut chewers, compared to those with more than 12 years of schooling respectively. Surprisingly, we have found that those with informal education (Maktab) were five times more likely to be betel nut chewers (AOR 5.06; 95% CI 4.69–5.45) than those with more than 12 years of schooling. By occupation, those were in the agriculture AOR 1.33 (95% CI 1.25–1.41); unemployed/retired AOR 1.10 (95% CI 1.03–1.18); housewife AOR 1.53 (95% CI 1.43–1.63); business owner AOR 1.48 (95% CI 1.40–1.56); and skilled/unskilled labor AOR 1.61 (95% CI 1.53–1.70), were more likely to be betel nut chewers than participants who were service holders. It was also observed that married participants were more likely to have two times higher (AOR 2.35; 95% CI 2.19–2.52) odds of betel nut chewing practice than unmarried participants. Smokers were also 1.8 times higher (AOR 1.87; 95% CI 1.82–1.93) odds of chewing betel nut than the non-smokers.

## Discussion

The study using a nationally representative data set revealed that the national prevalence of betel nut chewers in Bangladesh was 31.4%. This is similar to the findings of another study conducted in Bangladesh in 2012, which reported the prevalence of betel nut at 31%. In another study, the reported prevalence of betel nut was 25%^16,18^. In this present study, the correlates for chewing betel nut were: having no education; 1–5 years of schooling; 6–12 years of schooling; or informal education (Maktab); specific occupations (agriculture, business, unemployment, housewife, labour, and others); being married, and smokers. Those living in rural areas and participants from 25 to 44 years old, 45 to 64 years old, and ≥ 65 years were more likely to be betel nut chewers.

Our study observed that the odds of chewing betel nut increase in a positive linear relationship with age. This finding of our study was in agreement with the study conducted in Bangladesh by Flora MS et al. showing that betel nut chewing increased significantly from younger to elderly age groups, with a peak at around the 50–69 year age group^18^. Although another study conducted among Bhutanese adults by Wangdi et al. found that the prevalence and the odds of chewing betel nut increased to a certain age (64 years old), then it slowed down a bit^19^. This might reflect the sample differences among these surveys.

Informally educated (Maktab) and less educated participants were found to have higher odds of chewing betel nuts than highly educated participants. The higher odds of chewing betel nuts among participants with informal education could result from a traditional practice here in Bangladesh. This is similar to other published studies^18,20,21^. In Bangladesh, the practice of chewing betel nut can more frequently be observed among religious preachers (such as Imam and Maolana) as a cultural practice. Not knowing about the dangers of chewing betel nuts or a lack of health knowledge could be the cause. Education attainment has always been an essential factor in the growth of a nation’s health status and an individual’s health attitude.

Participants from rural areas were more likely to chew betel nuts than participants from urban areas. Several published studies reported similar associations between geographical area (rural/urban) and betel nut chewing practice^18,19^. We have generated a hypothesis that this could result from less educated people residing in rural areas. The absence of appropriate health knowledge and the inability to identify the harmful consequences of chewing betel nuts may have stemmed from the lower level of education. In addition, rural residents would have less access to valuable and essential information and knowledge regarding good health than urban dwellers.

Our findings also showed that skilled/unskilled labourers and participants in agriculture were more likely to be betel nut chewers than participants who were service holders. However, business owners and homemakers were also found to be 1.5 folds more likely to be betel nut chewers than service holders. These findings were consistent with other studies conducted in South Asian countries^18,23^.

In our population, we have found that smokers were likelier to be betel nut users than non-smokers. Several empirical findings reported a similar association between smoking status and chewing betel nuts^18,21,24,25^. Betel nut chewing practice can be observed by both male and female participants in Bangladesh as in other south Asian countries^16,26^. Most of the previous studies have found that female participants were associated with increased chewing of betel nuts than male participants^8,18,27,28^. In this study, we have also observed such incidence, but our findings had no statistical significance. Conversely, a study by Dorji et al. found that men were more betel nut chewers than women^24^. This situation could be the result of using different study samples as our study used a nationally represented data sample.

Some predictor variables, such as informal education (Maktab), showed wider confidence intervals, indicating variability in responses. This may be due to small subgroup sizes or heterogeneity in betel nut consumption patterns within certain demographic groups. Future research should use stratified sampling to minimize variability.

The greatest strength of this study is that the data we used to estimate the prevalence of betel nut chewing and its distribution pattern among Bangladeshi socio-demographics was from nationally representative data. Thus, the findings of this study can be used for national preventive strategies because chewing betel nut is widely practised in Bangladesh. To reduce consumption, Bangladesh can implement community-based awareness programs targeting vulnerable groups, along with school-based educational initiatives discouraging initiation among youth. Insights from successful South Asian intervention models should be adapted to Bangladesh’s context. Further research is needed to assess recent trends and evaluate policy effectiveness in addressing this public health concern.

## Limitation

Firstly, since betel nut use was self-reported, there is potential for underreporting due to social desirability bias. Future studies should incorporate biomarker validation methods, such as salivary or urinary arecoline testing, to improve accuracy. Finally, the questionnaire was not exclusively developed with a focus on betel nuts. We lack information on various dimensions, such as: "amount of betel nut chewed", “probable variations in chewing frequency over time”, and other aspects that could be of interest. Other health data, such as cholesterol or level of physical activities that may throw further insights into the effects of betel nuts, were also unavailable for the same reason.

## Conclusion

In conclusion, the current consumption rate of betel nuts is pushing Bangladesh towards a higher rank in the world. This study highlights the high prevalence of betel nut chewing in Bangladesh, particularly among older, rural, and lower-educated populations. Given the strong social and cultural acceptance of betel nut use, intervention strategies should integrate public health messaging with culturally relevant frameworks.

To reduce betel nut consumption, Bangladesh can implement community-based awareness programs targeting vulnerable groups, along with school-based educational initiatives discouraging initiation among youth. Insights from successful South Asian intervention models should be adapted to Bangladesh’s context. Further research is needed to assess recent trends and evaluate policy effectiveness in addressing this public health concern.

## Data Availability

The datasets used and/or analysed during the current study available from Professor SR Mashreky (mashreky@ciprb.org) on reasonable request.
